# TTEO (Things Talk to Each Other): Programming Smart Spaces Based on IoT Systems

**DOI:** 10.3390/s16040467

**Published:** 2016-04-01

**Authors:** Jaeseok Yun, Il-Yeup Ahn, Sung-Chan Choi, Jaeho Kim

**Affiliations:** IoT Platform Research Center, Korea Electronics Technology Institute, 25 Saenari-ro, Bundang-gu, Seongnam 13509, Korea; jaeseok@keti.re.kr (J.Y.); iyahn@keti.re.kr (I.-Y.A.); csc@keti.re.kr (S.-C.C.)

**Keywords:** smart spaces, smart objects, Internet of Things, oneM2M platforms, object identifier, IoT ecosystem

## Abstract

The Internet of Things allows things in the world to be connected to each other and enables them to automate daily tasks without human intervention, eventually building smart spaces. This article demonstrates a prototype service based on the Internet of Things, TTEO (Things Talk to Each Other). We present the full details on the system architecture and the software platforms for IoT servers and devices, called Mobius and &Cube, respectively, complying with the globally-applicable IoT standards, oneM2M, a unique identification scheme for a huge number of IoT devices, and service scenarios with an intuitive smartphone app. We hope that our approach will help developers and lead users for IoT devices and application services to establish an emerging IoT ecosystem, just like the ecosystem for smartphones and mobile applications.

## 1. Introduction

Building smart spaces has been a long-standing research area in computer and communication systems. As systems, in particular, embedded networked systems, have become more ubiquitous and integrated into everyday objects, they become the essential building blocks for smart spaces–*smart objects* (also called smart artifacts). Researchers have attempted to use them as sensors and actuators for monitoring and controlling their environment.

The widespread adoption and global diffusion of smartphones and wireless connectivity, including ZigBee, Bluetooth, Wi-Fi, and cellular networks, enables things in the world to be connected to each other and comprises a huge network of networks, called the Internet of Things (IoT) [1]. The IoT is expected to dramatically change the way people interact with smart objects. It is not difficult to find a creative product that connects to smartphones, e.g., Quirky Egg Minder [2], a smart egg tray that can track the number of eggs in refrigerators and tell users which one is the oldest.

However, the rapidly-growing IoT market is expected to suffer from a massive fragmentation problem due to the lack of standardized software platforms for IoT devices and services. For example, more than ten vendors have introduced Internet-connected, programmable smart thermostats relying on totally different network protocol and their proprietary platforms [3]. Such proprietary systems will make it difficult to interwork with each other and provide smart services in collaboration with each other. For example, an interoperable set of an alarm clock, toaster, and coffee maker would be able to prepare a fresh breakfast in wake-up time, provided the home appliances are built on a common software platform (even though developed by all different vendors).

This introduces the need for two kinds of IoT systems: a connectivity platform for the infrastructure domain and a smart service server for the interaction domain. The former should be standardized and is needed to enable IoT products to interwork with each other in a standard way, e.g., a globally accessible Web interface. The latter will be needed to allow developers to build new services for a set of IoT devices registered with the connectivity platform. Indeed, if the smart service server can provide home automation services between IoT products using a rule-based control system consisting of predefined templates (e.g., if-then rule statement) [4], it will be able to help users customize and configure their smart space dynamically, for example if the alarm clock is set for 7 a.m., then make the toaster and coffee maker work for the user to eat breakfast on time. Recently, an introductory paper by Heral and Tarkoma has also highlighted several of the most important aspects for building smart spaces in the IoT era [5], including IoT market defragmentation, support for IoT software platforms, programming models for programming smart spaces, and intuitive and effective user interaction with smart spaces, which echo our motivation in this paper.

Consequently, our main contribution can be summarized:

- Develop a standardized connectivity platform to address the fragmented IoT market,
- Develop a smart service server to customize and configure IoT products connected with the connectivity platform, and
- Develop a prototype service to demonstrate how to program smart spaces with IoT systems.

The details of the proposed approach are given as follows. First, we have developed IoT software platforms, called Mobius and &Cube, which can serve as connectivity platforms for IoT servers and devices, respectively. Both platforms are designed to be compliant with a globally-applicable IoT standard, oneM2M [6], ensuring that all devices embedded with the &Cube can be interoperated with each other via a suite of Representational State Transfer Application Programming Interfaces (REST APIs) provided by the Mobius on a global scale. Indeed, the platforms are architected to support Object Identifiers (OIDs), allowing IoT devices to be uniquely identified. This global standard-driven connectivity platform enables a wide range of IoT devices from even different manufacturers to interwork with each other, thus helping minimize the expected fragmentation problem of the IoT ecosystem. Second, we have developed a smart service server for configuring smart services, which consists of three main parts: a data container for retrieving data from the Mobius, a rule table for maintaining a list of if-then rule statements, and a rule engine for performing tasks described with if-then rules. Finally, we demonstrate a prototype service, TTEO (Things Talk to Each Other), which enables users to program smart spaces by setting if-then rules for several off-the-shelf IoT devices to automate daily tasks without user intervention.

From the perspectives of key players in the IoT ecosystem, including device manufacturers, service developers, and end-users, the proposed method is expected to help device manufacturers overcome the market fragmentation problem by supporting standard-based IoT platforms with global Web interfaces; enable service developers to create various smart services with the smart service server; and offer end-users greater flexibility of choosing IoT products, for example to select products from different vendors to organize a smart service as in the alarm clock-toaster-coffee machine scenario. However, we discuss future works for the evaluation study of the TTEO service and the remaining challenges for establishing a more expansive ecosystem by jointly working with other IoT-related standard bodies.

The remaining organization of the paper is as follows. Section 2 introduces smart objects and the IoT, and highlights open challenges and opportunities towards building smart spaces based on IoT systems. Section 3 presents our approach, including the system architecture, operation procedure, service scenario, and identification scheme. In Section 4, ecosystem analysis based on the proposed architecture is explained from the perspectives of three key players, including device manufacturers, service developers, and end-users. Section 5 describes remaining challenges and future works, and Section 6 offers concluding remarks.

## 2. Related Works

### 2.1. Smart Objects

It seems that smart objects have many different definitions depending on the application domains, but imply a physical or virtual entity that has the capabilities of sensing, communicating, processing and reacting to the external conditions of the environment. Since the term ubiquitous computing was coined by Weiser in the 1990s, smart objects have been seen as a key enabler of smart spaces, like the home and workplace [7,8]. In particular, the Disappearing Computer Initiative explored the use of interacting smart objects for enhancing our daily life [9]. For example, Strohbach *et al.* introduced cooperating smart objects based on an if-then rule-based inference chain and its case study, which explores the use of chemical drums alerting about potentially hazardous situations, programming the actions of everyday physical objects [10]. Siegmund introduced examples of context-aware communication services based on a communication platform for smart objects and embedded device platforms [11].

### 2.2. The Internet of Things

The term IoT, first coined by Ashton [12], has recently become popular, in particular in the scenario of modern wireless telecommunications. The IoT represents a technological revolution, where all of the objects in the real and virtual world can be connected to each other through the Internet due to several technological advances, including identification and contactless data exchange (RFID and NFC), distributed sensor networks, short-range wireless communication (ZigBee and Bluetooth) and universal mobile accessibility (Wi-Fi hotspots and cellular networks), sharing the information about the status change in their environment, eventually becoming smart, reactive to external stimuli, and collaborating as building blocks to create smart spaces [1,13]. With the current spotlight on the IoT, smart objects and their applications are recently getting more attention; for example, Kortuem *et al.* reported the potential of smart objects as building blocks for the IoT and novel computing applications [14]. Recently, Atzori *et al.* highlighted the opportunities of integrating social networking concepts into the Internet of Things, e.g., socially-interoperable smart objects [15].

### 2.3. Programming Smart Spaces

Wasik pointed out that the IoT revolution will make the world we are building “programmable” [16]. He claimed that the IoT revolution will happen in three main stages: (1) getting more objects onto the network; (2) programming their actions to carry out simple tasks without human intervention; (3) understanding connected objects as a single giant system to be programmed and building rich applications creating complex interrelationships among them. Considering ever-increasing advances in computing and communication technologies and the IoT-inspired emerging phenomena, like IFTTT (If This Then That) [17] and SmartThings [18] (see the details below), it seems that we are now experiencing both the first and the second stages of the IoT revolution. IFTTT is a service that allows users to connect various Internet-based application services (e.g., Facebook) by creating rules (called recipes) using simple if-then statements. SmartThings offers smart home automation services with the SmartThings Hub (*i.e.*, a smart gateway for connecting all compatible products together) and various home devices, including sensors, locks, light switches, electrical outlets, and thermostats. Recently, advances in Semantic Web technologies (in particular, linked data and linked rules) enable rules to be shared over the Web, and thus effectively reused and combined to help developers design cross-domain IoT applications [19,20].

### 2.4. Toward a Programmable World

Recently, IFTTT has been working on integrating digital services with smart products (e.g., SmartThings, Belkin WeMo Home Automation [21], Philips Hue LED light bulb [22]). This highlights two important aspects of a successful and widespread adoption of and a great user interaction and experience with IoT-based smart products for building smart spaces.

The first one is the use of a common IoT platform between different manufacturers. The smart products from either SmartThings or Belkin WeMo were originally and independently designed to work with their own proprietary platforms, hardware, and smartphone applications for monitoring and controlling them. However, by integrating with IFTTT, IFTTT works just like a common platform that enables users to tie smart products from different companies together, being able to bridge the gap between multi-brand home automation devices working in isolation. For instance, IFTTT provides recipes to connect SmartThings to the Belkin WeMo Switch [23]. A simple, graphical user interface for creating and sharing recipes is also one of advantages of the IFTTT integration.

Another important aspect of the IoT-inspired solutions is the use of open APIs. One example is Philips Hue, the Internet-connected LED light bulb. It involves a radio chip and small computer storage, so that we could control the LED light bulb via a smartphone application. Surprisingly, in 2013, Philips released its open APIs and software development kit (SDK) for iOS developers (now also available for Android) and guides for both hardware and software makers [24]. Now, it is possible to see more than 100 iOS apps on Apple’s App Store (even more Android apps on Google Play) working with Hue created by all different developers. This indicates that open API-based IoT solutions might be able to open up great revenue opportunities for both hardware and software developers, but also to give users a chance to experience various products and services at reasonable costs.

Recently, the new software platforms of iOS 8 unveiled in 2014, HealthKit and HomeKit, also imply that Apple will unlock their systems to developer communities so that a wide range of device makers and application programmers could create innovative products and services working with Apple’s products in healthcare and smart homes that leverage Apple’s ecosystem.

As these examples demonstrated, the key features for a programmable world are a common infrastructure for supporting the development and distribution of smart objects, data, and applications and a user-friendly interaction method for supporting the deployment and configuration of smart objects. While the former aims at lowering barriers for device makers and application developers to create and distribute IoT-based devices and applications, the latter aims at fostering end-users to deploy and configure smart objects without any technical or programming knowledge. This idea is inspired by previous studies discussing the importance of developer/user support for the distribution and sharing of IoT artifacts based on connected marketplaces [25,26]. The remarkable efforts of researchers in the ubiquitous computing [27,28,29] and the Web of Things (WoT) communities [30,31,32] to provide end-users with a way to build context-aware systems with no programming skill also echo our motivation in this research.

## 3. Our Approach

Helal and Tarkoma summarized open research questions on building smart spaces: the design and construction of smart spaces to overcome the fragmented markets; architecture, middleware, and programming models to program smart spaces dynamically and effectively; seamless coexistence of a smart space with the ubiquitous Web; support for providing space-specific impromptu services; intuitive user interaction with smart spaces [5]. In Table 1, we briefly summarize how our approach could answer the research questions, since they are substantially aligned with the motivation for this work. The IoT platforms driven from global standards (*i.e.*, oneM2M and OID) will allow smart objects from different manufacturers to be connected to each other, finally minimizing the fragmented market. In addition, globally-accessible Web interfaces offered with REST and Message Queuing Telemetry Transport (MQTT) APIs will drive more smart objects to be easily integrated with the ubiquitous Web. The TTEO prototype service based on the TTEO server, the GUI-based app, and tangible devices (e.g., smart dice) could help end-users dynamically and intuitively program smart spaces, *i.e.*, customize and configure smart objects to perform daily tasks. The full details are described below.

### 3.1. Proposed Architecture

**Figure 1:**
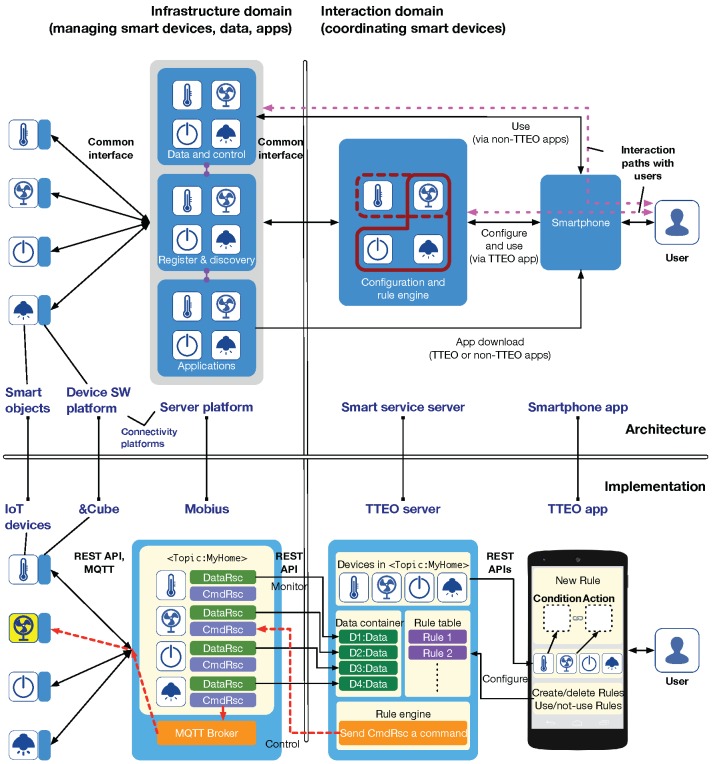
System architecture and implementation for IoT-based smart services.

The top of Figure 1 shows the system architecture for IoT-based smart services, where the left side represents the infrastructure domain and the right side represents the interaction domain.

In the infrastructure domain, there are three sub-systems: smart objects, device software platform, and server platform. The device software platform is the foundation of smart objects on which various applications run and provides Internet connectivity to server platforms via common interfaces. Server platforms maintain virtual entities representing smart objects in the real world and allow end-users to interact with them, e.g., look-up, monitoring, and control. Accordingly, they are required to provide three main functionalities: registering and discovering smart objects, collecting data from and sending commands to objects, and managing applications for objects and smartphones. In the interaction domain, there are two ways to provide smart services for end-users, drawn as dotted lines at the top of Figure 1. The first one is along the direct path between smart objects (*i.e.*, virtual entities in server platforms) and smartphone apps. The other one enables end-users to configure smart services dynamically and effectively in smart service servers through smartphone apps specifically developed for the services, e.g., home automation. One advantage of this method is that it may allow end-users to select a service provider that provides the best service. Thus, we focus on the second interaction method via a service server.

### 3.2. Our Implementation

There are five essential systems for providing smart services in our architecture: smart objects, device software platform, server platform, smart service server, and smartphone apps. Accordingly, a prototype system comprising IoT devices, &Cube, Mobius, TTEO server, and TTEO app is implemented as shown at the bottom of Figure 1.

#### 3.2.1. IoT Devices

**Figure 2:**
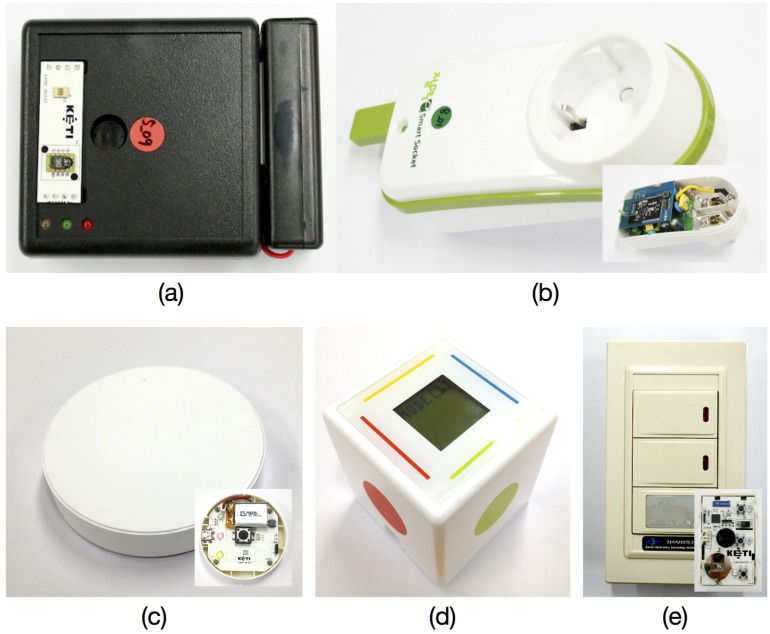
Prototype IoT devices: (**a**) multi-functional sensor for measuring temperature, humidity, illumination, and occupancy; (**b**) smart plug; (**c**) wireless switch; (**d**) smart dice; and (**e**) wall light switch.

IoT devices could be any device that will be connected to our server platform. We assume that IoT devices are located within wireless personal area networks (e.g., ZigBee) and wirelessly connected to an IoT gateway (explained in detail in Section 3.2.2), in turn linked with the server platform. Specifically, three categories of IoT devices are implemented, sensors, smart plugs, and user input devices, as shown in Figure 2.

We first developed prototype multi-functional sensors incorporated with a temperature sensor, humidity sensor, illumination sensor, and PIR (Pyroelectric Infrared) sensor (Figure 2a). Next, smart plugs are developed, which can be used as both sensors and actuators (Figure 2b). A smart plug can measure electrical power consumed in an electrical device plugged in and instantly turn the device on or off. The full details on the design and implementation of the smart plug are described in our previous literature [33]. Finally, for an easy-to-use, user-friendly input interface, wireless switches (Figure 2c) and smart dice (Figure 2d) are developed. Wireless switches command smart plugs to turn electrical devices on or off, just like a remote controller. The smart dice is embedded with a three-axis accelerometer and provides four different inputs by positioning each sensitive axis (X, Y) of the accelerometer at +g and -g, each of which can command smart plugs or other devices to perform a task. Users could also change the mode of the dice with simple shaking gestures and assign another set of four commands to devices. The dice has a small LCD panel that shows its mode and the description of the four commands assigned, e.g., “turn a fan on”. For wireless wall light switches (Figure 2e), we have modified a commercially-available IR remote control wall light switch to be connected to our IoT gateway. All of the IoT devices developed use the Texas Instruments SoC solution for IEEE 802.15.4 applications and CC2530 to provide wireless connectivity to the IoT gateway.

#### 3.2.2. &Cube

The &Cube is a middleware platform running on Linux-based embedded machines and performs two main functions. The first one is to transmit data collected from IoT devices to our server platform and to send command messages to other IoT devices, like smart plugs. The second one is to download and run device applications for the operation of IoT devices registered into the &Cube. The &Cube is designed to be compliant with oneM2M standards that define common M2M/IoT service layer specifications for globally-applicable, access-independent M2M/IoT solutions [34]. A more thorough explanation of the oneM2M standards is out of scope and available on the oneM2M homepage [35].

**Figure 3:**
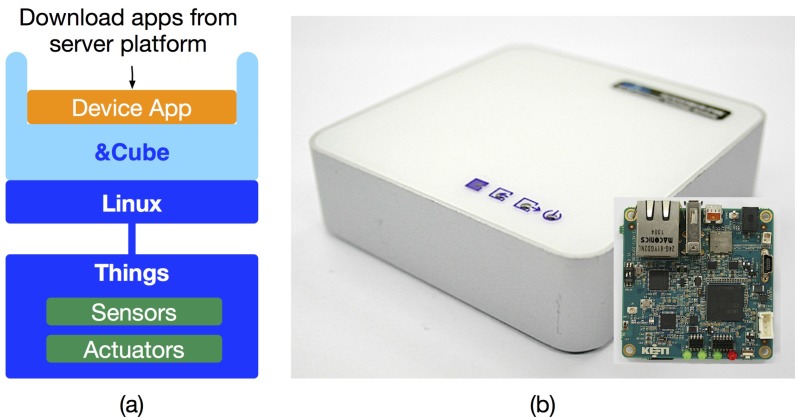
The &Cube and IoT gateway: (**a**) architecture of our device software platform (&Cube) and (**b**) the IoT gateway developed (IoTG100) on which the &Cube is running.

From the oneM2M perspective, the &Cube can serve as a Middle Node (MN) and Application Service Node (ASN). Besides such standardized aspects, we have also designed the architecture of the middleware platform in such a way that newly-released device applications can be downloaded from our server platform according to end-user’s requests, as shown in Figure 3a. For example, it is reasonable to imagine a laundry machine embedded with the &Cube, which can automatically download its device apps having new washing features. Details on the architecture and design principles of the &Cube are described in our recently-published literature [36].

For an IoT gateway, we have developed the IoTG100 consisting of a Samsung S5PC210 Exynos 4 Dual 4210 SoC, 1 GByte memory, 10/100-Mbps Ethernet connector, 2.4-GHz IEEE 802.15.4 ZigBee transceiver, Wi-Fi and Bluetooth module, and SD card socket, as shown in Figure 3b. It runs the Linux kernel for embedded systems and Java SE Embedded, on which our Java-based &Cube operates, and provides wireless connectivity, including Wi-Fi, Bluetooth, and ZigBee for IoT devices.

#### 3.2.3. Mobius

The Mobius is an IoT server platform complying with the oneM2M standards. It provides all of the functionalities for IoT devices, including registration, data management and repository, device management, security, communication management and delivery handling, discovery, subscription and notification, *etc*. For interconnecting the Mobius and a large number of &Cubes, the Mobius provides bindings for HTTP and MQTT (Message Queuing Telemetry Transport). Recently, Ryu *et al.* presented a prototype smart office implementation, which can provide smart services by controlling office utilities registered with the Mobius according to the user’s situation [37].

The Mobius provides two important capabilities for managing IoT device resources: data acquisition (presented as DataRsc in Figure 1, a sort of data storage resource archiving data for IoT devices), and command control (presented as CmdRsc in Figure 1, a resource for controlling the operation of IoT devices). With the assumption that a sensor and actuator are registered with the Mobius via the &Cube, we can obtain data collected from the sensor using REST APIs offered by the Mobius. We can also control the actuator by modifying its corresponding CmdRsc in the Mobius, and eventually, its MQTT broker will send the command to the device.

From the oneM2M perspective, the Mobius can serve as an Infrastructure Node (IN). Besides such standardized aspects, the Mobius provides group management of IoT devices by grouping them into the same “Topic”, which can be used as a key to look up a list of IoT devices related to a given service. For example, <Topic:MyHome> represents a group of IoT devices related to the MyHome topic for a home automation service. The Mobius also provides an app store that distributes application software running on the &Cube (*i.e.*, device apps) and smartphone apps (or links to Web apps) on which users can interact with IoT device-based smart services.

#### 3.2.4. TTEO Server and App

Although the Mobius provides all of the functionalities for device registration, data collection, and device control, it is a general-purpose IoT server platform for Internet connectivity. Thus, it is additionally required to build a service server that is responsible for configuring smart services. In the TTEO service, the main task of a service server is to perform rule-based control of the devices grouped into a Topic based on the information collected from the IoT devices. Rules are created using the TTEO app, a GUI-based smartphone app [38], which is designed to allow users to intuitively and dynamically configure rules between IoT devices in smart spaces.

**Figure 4:**
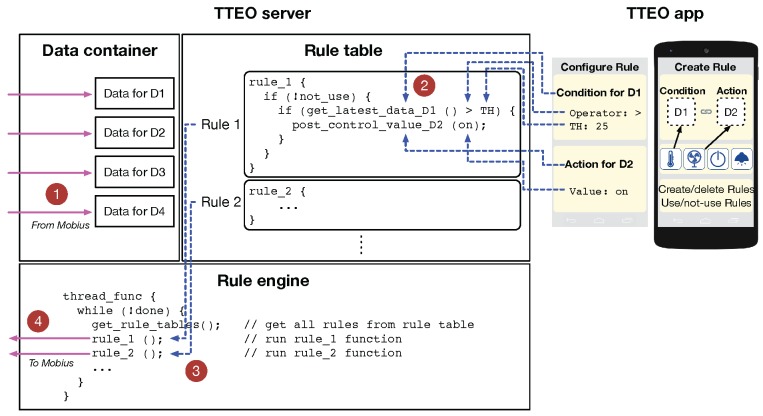
TTEO service server operation: create rules and run its rule engine.

Figure 4 (*i.e.*, bottom right part of Figure 1) illustrates technical details of TTEO server operations with the TTEO app for creating new rules and running its rule engine for smart services. Once a user has logged in to the TTEO server with a specific Topic, the TTEO server takes the list of the IoT devices grouped in the Topic the user has registered and then retrieves the data of the devices from the Mobius at a regular interval, as shown in the circled number “1” of Figure 4. The TTEO server also manages a rule table consisting of a list of if-then rule functions for smart services (*i.e.*, if-then statements in Java codes), as shown in the circled number “2”. When constructing each rule function, the methods for retrieving data (e.g., get_latest_data_D1()) or controlling devices (e.g., post_control_value_D2()) are automatically overloaded according to the type of devices chosen by the user via the TTEO app (see Section 4.1 for more details on the methods). Furthermore, the relational operators (e.g., >) and parameters (e.g., TH, on) are plugged into the rule function. Finally, the rule engine creates a thread function for the user, where the list of rule functions created by the user is loaded and sequentially run (see the circled number “3”). When running each rule function, if needed, the rule engine sends a command to the CmdRsc in the Mobius for the device designated to perform a task (see the circled number “4”).

### 3.3. Operating Procedure and Use Scenario

**Figure 5:**
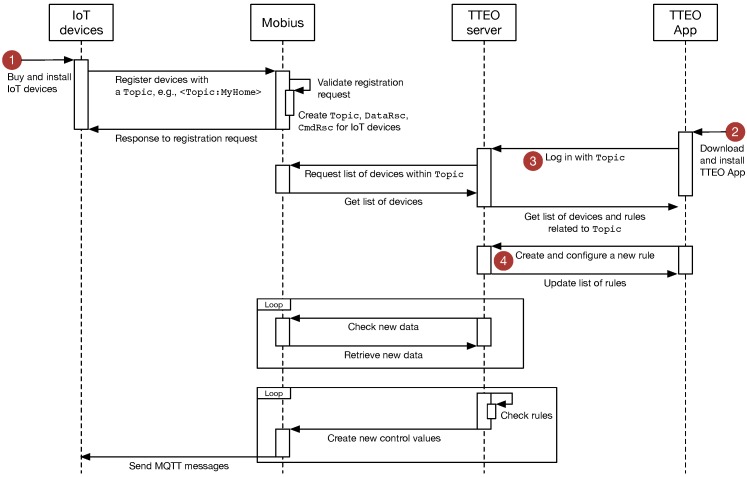
A sequence diagram for operating procedure of TTEO service.

In this section, the operating procedure and use scenario for TTEO service is described based on the implemented systems. Figure 5 demonstrates a sequence diagram for TTEO service, where the circled numbers 1 through 4 are specifically described in the below steps.

**Figure 6:**
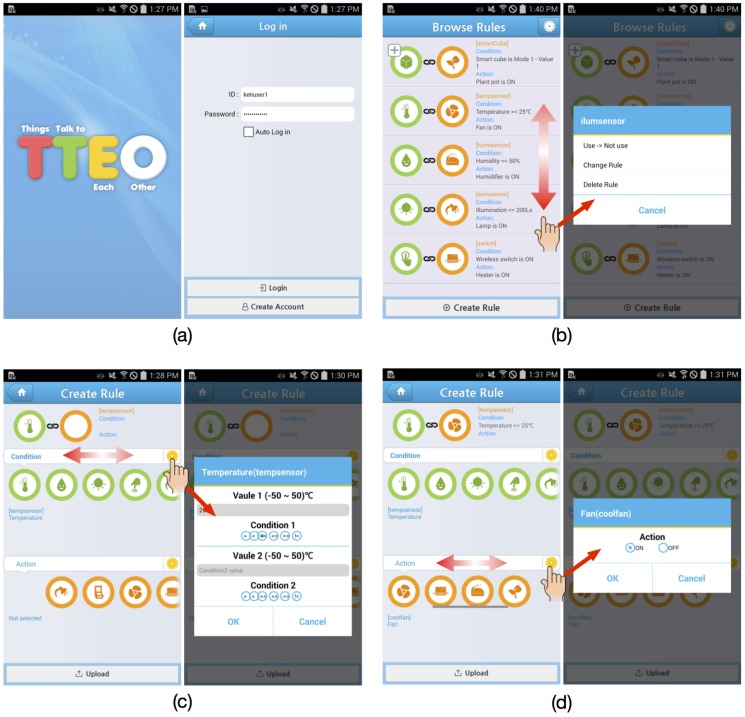

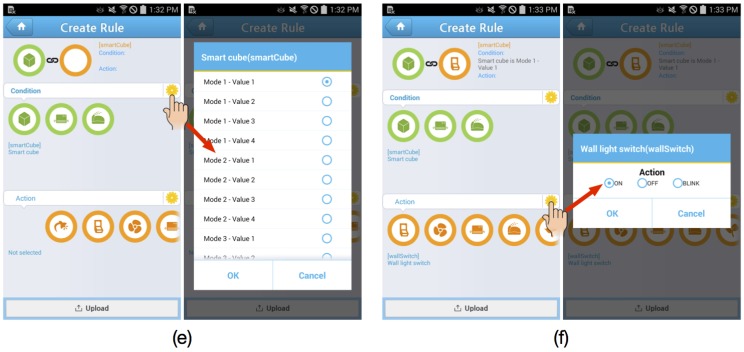
TTEO app examples: (**a**) open and log-in; (**b**) browse rules and configure each rule; (**c**) create a rule between a thermometer and fan-condition setting; (**d**) create a rule between a thermometer and fan-action setting; (**e**) create a rule between smart dice and a wall light switch-condition setting; (**f**) create a rule between a smart dice and wall light switch-action setting.

- Step 1
  Choose an IoTG100 and IoT devices users want to install in smart spaces and register them into the Mobius with appropriate descriptions and a specific Topic, e.g., <Topic:MyHome>. It could be expected that users might be able to purchase devices on offline or online stores in the near future. Furthermore, consumer electronics manufacturers may reflect the demand for open API-based smart services and plan to launch home appliances that could be controlled using open APIs. Once registered, systems will automatically create DataRsc and CmdRsc for the new devices.
- Step 2
  Download the TTEO app directly from Google Play or the Mobius app store and run it. Figure 6 presents the captured images of the TTEO app.
- Step 3
  Create a new account and log-in (see Figure 6a). For simplicity, in this prototype version, it is assumed that a single service provider runs both Mobius and TTEO server, so that users can use the same ID and password they already registered in Step 1, or otherwise, users need to create a new account for the TTEO. Once logged in, the TTEO server takes the list of all devices within Topic and data from the Mobius. Furthermore, users can browse the rules if previously created (see Figure 6b). With a vertical swipe of the finger across the screen, all of the rules can be browsed. Tapping a rule on the screen opens a pop-up window, which enables users to decide if the rule will be used, modified or deleted.
- Step 4
  Create a new rule (Figure 6c–f). The screen consists of three rows and an upload button. At the top row, the left side illustrates the rule just created as an icon view, while the right side describes details about the rule, such as the parameter values. Both sides will be automatically modified as users further change the rule setup below. The second and third rows each represent the condition and action terms that corresponds to an if-then statement. IoT devices are displayed with a set of predefined icons according to their categories, so that users can switch with a horizontal swipe of the finger and intuitively select a device with which they want to organize rules. In case of more than two devices registered in the same category, users distinguish between them using the description entered in Step 1.

After selecting devices in the condition and action terms, it is necessary to click the yellow gear icons on the right end and to configure additional information depending on the chosen devices. Table 2 summarizes the data type and operators used in conditional statements when creating rules. For example, in the case of selecting the temperature sensor, it is necessary to enter a specific operator (e.g., ≥) and float value in degrees (e.g., 25 ${}^{\circ }$C), as shown in Figure 6c. Recall that these operator and value will be plugged in the rule function as described in Figure 4. In case of selecting smart dice, we have a different configuration window popped up to select the mode and value as one of sixteen pairs of integer values of one to four, as shown in Figure 6e. For actions, the current TTEO system provides two type of devices: smart plugs and a wall light switch. For example, in the case of selecting home appliances plugged into smart plugs, it is necessary to enter on or off, as shown in Figure 6d. In the case of wall light switches, the config window shows three options for action: on, off, blink. In Figure 6f, a wall light switch is set to on if the smart dice gives Mode 1-Value 1.

One important issue when constructing a rule-based control system is to perform verification of the rule sets users have created, e.g., rule correctness or conflict. For this purpose, a simple rule verification procedure when creating a new rule has been implemented in the TTEO app. In case of sensor devices having a float data type, the TTEO app is designed to reject the input values that are out of the acceptable range for a given device, which will be provided by device manufacturers. Input devices are set such that only a single value (e.g., one of sixteen options for the smart dice) can be chosen using a mutually exclusive check box. In order for users to avoid creating rules that will cause smart plugs to work unexpectedly (e.g., two rules trigger the action of one smart plug at the same time), the TTEO app allows users to create only a single rule per smart plug as an action. Of course, as rules become more complex and complicated, a well-designed verification method in the TTEO server will be clearly required to validate the completeness and correctness of the rule datasets, as demonstrated in the rule-based expert system [39]. For example, a simple way for verifying rule-based systems is to build a decision table that enumerates all combinatorial decision rules based on conditions and actions and eliminates redundancy and inconsistency among decision rules, e.g., a set of specific rules to a given IoT device.

**Figure 7:**
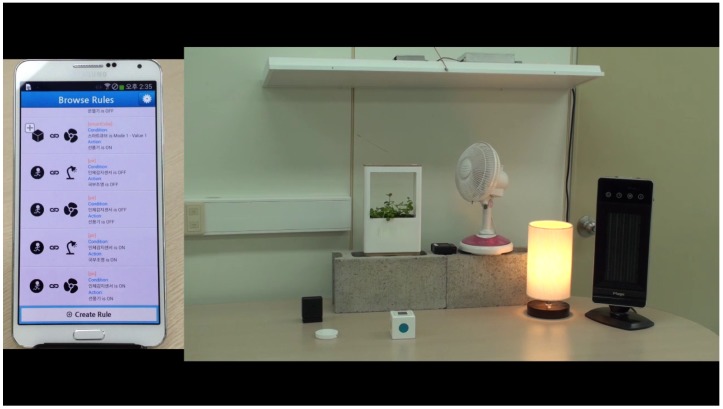
A captured image of the TTEO service scenario.

Figure 7 presents a captured image of the service scenario, and the full demo video is available on YouTube [40].

### 3.4. IoT Device Identification and Security

**Figure 8:**
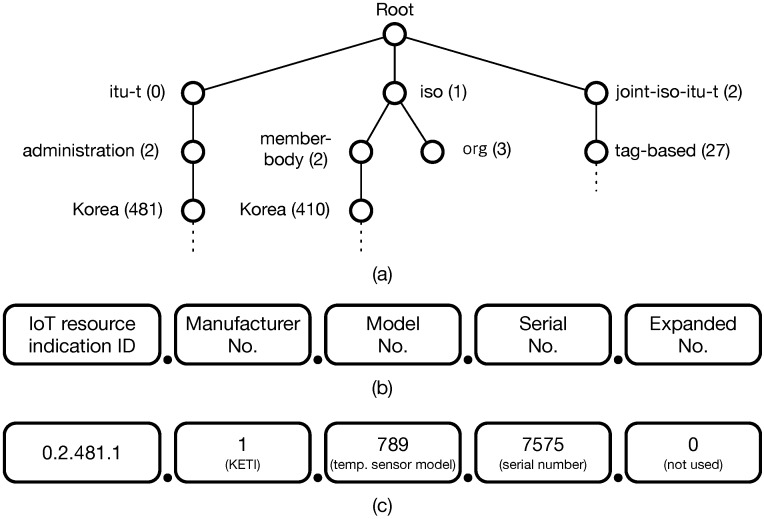
The OID-based IoT resource identification structure: (**a**) the OID hierarchy, (**b**) the proposed ID structure for IoT devices, (**c**) an OID example of the temperature sensor whose manufacturer is KETI, model number 789, and serial number 7575, provided the root for IoT resource indication in Korea is 0.2.481.1.

The TTEO service can be realized assuming that systems can uniquely identify all devices, just like MAC addresses for network devices. Accordingly, we consider a globally-unique identification scheme for IoT devices based on the Object Identifier (OID). The OID consists of a set of nodes, each of which is hierarchically assigned from its parent node with successive numbers separated with dots, e.g., 0.2.481.⋯. Standard authorities, such as ISO (International Organization for Standardization) and ITU-T (International Telecommunication Union-Telecommunication standardization sector) issue the root of the OID tree (0, 1, 2), as shown in Figure 8a. Each node in the tree can be identified by communicating with OID repositories and resolution servers managed by manufacturers. Figure 8b presents the proposed ID structure for IoT devices showing IoT resource indication ID at first followed by information about the manufacturer, model, serial, and reserved node for expansion. Figure 8c illustrates an OID example. We assume that a standard authority may issue the root OID for IoT devices in Korea, *i.e.*, 0.2.481.1. With this reasonable assumption, the example shown in Figure 8c represents a temperature sensor whose manufacturer is KETI; the model number is 789; and the serial number is 7575.

This identification scheme requires a process of standardization, involving a wide array of players across multiple industries. Accordingly, we have been working on activities to standardize the scheme and architecture for the IoT devices and services presented, finally being included in the oneM2M technical specifications [41].

Another big concern for building smart spaces based on IoT systems is security. Open connectivity among IoT devices in the world may open the gate to give malicious hackers the opportunity to record our sensitive data or to control our private machine at home. Accordingly, to make smart services using the oneM2M platforms and IoT devices secure, we have used an access token and user key issued by an authentication server, each of which encapsulates the security identity of a given IoT device and user, respectively. HTTPS has also been employed on the Mobius and TTEO service server to ensure the secure transmission of data.

## 4. Ecosystem for IoT Device Manufacturers, Service Developers, and End-Users

A newly-emerging IoT ecosystem is expected to be established by key players, including device manufacturers, service developers, and end-users. In the case of IFTTT recipes working with Philips Hue, Philips is the device manufacturer, and IFTTT allows service developers to create new services (e.g., recipes triggered by SmartThings presence detectors) on its common platform with APIs offered by Philips. However, this ecosystem is fairly limited to the IFTTT common platform. When creating new devices, the manufacturers need to do integration with the IFTTT platform, but also with other IFTTT-like platforms in expectation of an expansive IoT ecosystem.

**Figure 9:**
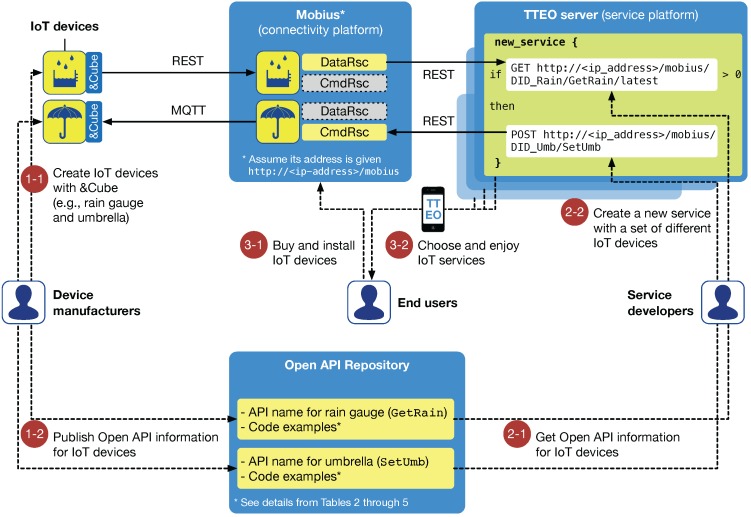
Ecosystem analysis of our approach from the perspectives of device manufacturers, service developers, and end-users.

In our approach, the common platform is independently divided into the connectivity platform (*i.e.*, Mobius) and service server (*i.e.*, TTEO server). We expect this to contribute to establishing a more expansive IoT ecosystem. Figure 9 illustrates the ecosystem analysis of our proposed approach from the perspectives of three main key players, including device manufacturers, service developers, and end-users. Here, we assume that the Mobius and TTEO server are basically supported by a network operator and service provider, respectively.

### 4.1. Device Manufacturers

Specifically, as shown in the circled number “1-1” of Figure 9, device manufacturers create IoT devices based on the oneM2M-compliant device software platform, &Cube (e.g., rain gauge and LED-embedded umbrella). This enables them to be connected to the Mobius, as well as any other oneM2M-compliant server platforms (e.g., SK Telecom “ThingPlug” [42] or InterDigital “oneMPOWER” [43]) in a standardized way, which naturally implies that the new devices will be able to be seamlessly deployed in any IoT services based on oneM2M standards.

At the same time, the manufacturers can publish open API information for their devices on the Internet, as displayed in the circled number “1-2” of Figure 9. We have built a Web portal, called the Open API Repository [44], for sharing the information about open APIs for IoT devices. Similar to the Philips Hue developer community [24], the Open API Repository is proposed to serve as a collaborative developer hub for all oneM2M-compliant IoT devices and their open APIs. In our example, GetRain and SetUmb are published as open APIs, each of which can be used to retrieve the latest rainfall value measured from the rain gauge and to turn on or off the LED light of the umbrella, respectively.

Table 3, Table 4, Table 5 and Table 6 summarize code examples for the request and response of the GetRain and SetUmb APIs. In the tables, all necessary information (e.g., mandatory XML elements in the response body) is not specifically presented due to space limitation. Table 3 describes that to get the latest rainfall value, use HTTP verb GET followed by the full address of the data container of the rain gauge, http://<ip_address>/mobius/DID_Rain/GetRain/latest, where <ip_address>/mobius will be given by its network operator, and DID_Rain is issued by standard authorities, as explained in Section 3.4. Table 4 summarizes the code example of the response to the GetRain request. Once the request is fully approved, the Mobius sends back the 200 OK status with the XML body holding the latest rainfall value, e.g., 100 mm/h. Similarly, Table 5 presents the code example for SetUmb with HTTP verb POST followed by the full address of the control container of the umbrella, and appropriate XML body with a Boolean value representing LED-on. Table 6 summarizes the response example to the SetUmb request.

### 4.2. Service Developers and End-Users

Once device manufacturers have created IoT devices and published their API information in the Open API Repository, a service developer could get the information and create a new smart service working for the devices (*i.e.*, similar to an IFTTT recipe): if it’s raining then turn on the umbrella LED; as illustrated in the circled numbers “2-1” and “2-2”. Another developer could also develop more advanced services by integrating with public open APIs for weather forecast. End-users purchase and install IoT devices on the Mobius (see “3-1”), and will be able to choose the best service they would like to use (see “3-2”). Consequently, all of the scenarios in the IoT ecosystem are based on the standardized connectivity platforms and smart service servers, and thus, our approach might be able to provide better revenue opportunity for device manufacturers and service developers, and greater flexibility of choosing IoT products for end-users.

## 5. Discussion

In this section, the advantages of the proposed approach are demonstrated, compared to the commercial IFTTT service working with SmartThings. Furthermore, we discuss future works and remaining challenges to build smarter spaces based on IoT systems.

### 5.1. TTEO vs. SmartThings + IFTTT

The TTEO service has two main advantages compared to other commercially available services, such as SmartThings combined with IFTTT.

**Figure 10:**
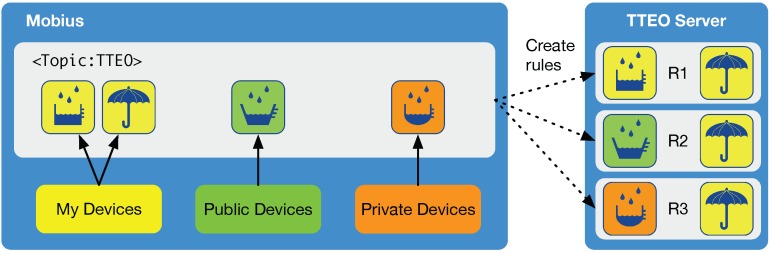
Create rules using my devices, public devices and private devices.

From the point of view of usability, the first advantage is to allow users to organize rules using the devices installed in their smart spaces, as well as other devices installed in public spaces and even in private spaces by paying a service fee. For example, as shown in Figure 10, we can imagine a scenario where a rule R1 can be set: if the rain gauge in our home landscape detects rainy in the morning, then the umbrella let us know “it’s rainy” by a glowing light or beeping. Rules R2 and R3, shown in the figure, demonstrate that it will also be possible to get the smart rain alarm service by setting rules with either a public or private one installed near the house. Most of the public and private devices might be likely to be sorts of sensors, but not physical actuators, which cannot be used at the same time.

From the point of view of the open API- and common platform-based development framework, another advantage of the TTEO service will be being able to help establish an ecosystem for device makers, service developers, service providers, and end-users. In fact, most companies, such as SmartThings, develop their proprietary platforms, products and mobile applications and further look for ways to integrate directly with products manufactured by other companies, e.g., connecting SmartThings to Philips Hue. However, our development framework is designed to help companies stay more focused on what is truly important, *i.e.*, creating innovative IoT products.

### 5.2. Build Smarter Spaces

Although the current prototype TTEO service lets users create rules to perform a single task for devices, *i.e.*, power on/off via smart plugs, we could further promote this idea by allowing users to set device-specific configuration, e.g., dimming control of a lighting device, provided that it runs on our platforms and its open APIs for dimming control are published in the Open API Repository. Organizing rules is similar to creating programs using programming language, and thus, we may also provide a way to build multi-condition and multi-action rules by employing comparison and logical operators in conditional statements and if-then-else statements. Device scheduling will also be an important part to automate device management and operation.

Another key technology for building smart spaces is to give devices the ability to learn, infer, make decisions, and adapt their behaviors–becoming smarter. One common example is the Nest learning thermostat [45], a smart thermostat that can learn the occupant’s schedule and heating/cooling behavior, program itself, and be controlled from mobile applications. Similarly, given an IoT-based thermostat and its open APIs, we will be able to implement an intelligent agent in a TTEO server based on machine learning technologies, as in [46], which will work similar to the Nest thermostat. In addition, it is possible to further enhance the performance of the smart thermostat by taking advantage of complementary information about changes in the physical environment, including room-level temperature and occupancy, as well as by providing means of controlling another Internet-connected devices, like room fans.

Nevertheless, the human factors and usability study should remain at the core of all of the aforementioned ideas. Note that technological advances, such as IoT, may not only help us make the world smarter, but also make things more complex from the users’ perspectives. Accordingly, an evaluation study of TTEO services should be performed for end-user perspectives. For example, people may feel uncomfortable when dealing with dozens of if-then rules, and thus, it will be required to construct rules using spatiotemporal relationship-based actions or environment personalization with a simple, intuitive, and easy-to-use user interface, as discussed in prototyping context-aware applications [29]. As a result, we expect this evaluation study of TTEO services for our future work, considering *how smartly and efficiently* users can build smarter spaces.

### 5.3. Interoperability with Different IoT Standards in the Service Layer

The oneM2M initiative is developing *de facto* standards for creating globally-applicable, access-independent IoT applications and services. However, there are two main industrial alliances, AllSeen Alliance and Open Interconnect Consortium (OIC), each of which is developing its own specifications that allow developers to easily create IoT applications and services on the top of their framework, AllJoyn and IoTivity, respectively. Google’s Brillo/Weave has also been gaining attraction within IoT developer communities, but was excluded in our discussion because the details have not been specified yet.

To minimize the standards market fragmentation, the oneM2M is working on two Work Items (WIs) for interworking with AllJoyn (WI-0018) and OIC (WI-0044). These WIs are investigating a *standardized* way of interworking between oneM2M and AllJoyn systems or between oneM2M and OIC systems. For example, Yun *et al.* have presented interworking scenarios between oneM2M and AllJoyn devices (e.g., LIFX light bulbs [47]) by implementing a suitable Interworking Proxy Entity (IPE) for AllJoyn platforms [48]. More recently, a demo of interworking oneM2M and OIC systems (e.g., Samsung Smart TV and refrigerator) was presented at the 2016 Consumer Electronics Show, where our team contributed to the development of the IPE for OIC platforms [49].

Accordingly, these collaborative efforts will enable IoT developers and end-users to be free to choose their proprietary products independent of IoT standards. From the perspective of the TTEO system architecture, AllJoyn and OIC devices can be transparently mapped into oneM2M platforms (e.g., Mobius and &Cube), which in turn, are accessible and available via TTEO services. Consequently, TTEO users will be able to create rules for autonomous operations within a set of IoT products based on different standards, including oneM2M, AllJoyn, and OIC, even with other promising proprietary communications platforms (e.g., Google Weave) by developing appropriate standardized IPEs.

## 6. Conclusions

As many researchers highlighted in existing literature, common platforms and open APIs are considered as key enablers for establishing an IoT ecosystem without a market fragmentation. In this paper, we have proposed the IoT software platforms, Mobius and &Cube, complying with the globally-applicable IoT standards, oneM2M. For the widespread adoption of oneM2M standards and platforms, we have decided to make open source the code of the standardized part of the Mobius and &Cube under the OCEAN (Open allianCE for iot stANdard), an open source-based global partnership for IoT [50]. Developers can freely download, modify and share their work under the BSD 3-clause license [51]. We have partnered with IoT device manufacturers and service developers interested in our platforms and TTEO service. We are also working with university students who want to develop creative products and services using our platforms. We envision that the proposed development and service framework based on the IoT technologies will eventually be able to contribute to establishing a global ecosystem where all devices, products, applications, and services can meet with customers who want to build smart spaces.

## Figures and Tables

**Table 1:** Summary of research questions on building smart spaces and our approach.

Questions	Our approach
Fragmented IoT markets	oneM2M platforms (Mobius and &Cube),
	OID-based device identification scheme,
	Open API Repository, app store for IoT devices
Dynamic programming of smart spaces	TTEO server and smartphone app
Seamless integration with the Web	REST APIs and MQTT messages
Space-specific impromptu services	If-then rules setup and rule-based control
Intuitive user interaction	GUI-based configuration, smart dice use

**Table 2:** Data type and operators for IoT devices used in the conditions.

Devices	Sensing Data	Data Type	Operators Used in Condition
Sensor	Temperature	Float	==,>,<,≤,≥
	Humidity	Float	==,>,<,≤,≥
	Illumination	Float	==,>,<,≤,≥
	Electricity consumption	Float	==,>,<,≤,≥
	Occupancy	Boolean	==,!=
Input	Wireless switch	Boolean	==,!=
	Smart dice	16 pairs of integers (1–4)	== (chosen mutually exclusively)

**Table 3:** An example of the GetRain API request to retrieve the latest value measured.

GetRain API Request	
Method	**GET**
URL	http://<ip_address>/mobius/DID_Rain/**GetRain/latest**
Header	Accept: application/xml
	X-M2M-RI: 1234 (request id)
	X-M2M-Origin: origin_user (requester id)

**Table 4:** An example of the GetRain API response of which the body includes the rainfall value (100 mm/h).

GetRain API Response	
Status	**200 OK**
Body	<?xml version="1.0" encoding="UTF-8" encoding="UTF-8" standalone="yes"?>
	<m2m:cin
	xmlns:m2m="http://www.onem2m.org/xml/protocols"
	xmlns:xsi="http://www.w3.org/2001/XMLSchema-instance"
	rn="cin-20160203045942189Je30">
	<ty>4</ty>
	⋯
	**<cnf>rainfall</cnf>**
	**<con>100 mm/h</con>**
	</m2m:cin>

**Table 5:** An example of the SetUmb API request to turn the umbrella light on.

SetUmb API Request	
Method	**POST**
URL	http://<ip_address>/mobius/DID_Umb/**SetUmb**
Header	Accept: application/xml
	Content-Type: application/vnd.onem2m-res+xml; ty=4
	X-M2M-RI: 1234 (request id)
	X-M2M-Origin: origin_user (requester id)
Body	<?xml version="1.0" encoding="UTF-8" encoding="UTF-8"?>
	<m2m:cin
	xmlns:m2m="http://www.onem2m.org/xml/protocols"
	xmlns:xsi="http://www.w3.org/2001/XMLSchema-instance">
	**<cnf>boolean</cnf>**
	**<con>true</con>**
	</m2m:cin>

**Table 6:** An example of the SetUmb API response showing that a new resource (*i.e.*, control value) was created successfully.

SetUmb API Response	
Status	**201 Created**
Body	<?xml version="1.0" encoding="UTF-8" encoding="UTF-8" standalone="yes"?>
	<m2m:cin
	xmlns:m2m="http://www.onem2m.org/xml/protocols"
	xmlns:xsi="http://www.w3.org/2001/XMLSchema-instance"
	rn="cin-20160203045942189Je30">
	<ty>4</ty>
	⋯
	**<cnf>boolean</cnf>**
	**<con>true</con>**
	</m2m:cin>
